# Polysaccharide of *Atractylodes macrocephala* Koidz alleviates LPS‐induced proliferation, differentiation inhibition and excessive apoptosis in chicken embryonic myogenic cells

**DOI:** 10.1002/vms3.1412

**Published:** 2024-03-20

**Authors:** Mengsi Fu, Jinhui Wang, Danning Xu, Nan Cao, Wanyan Li, Fada Li, Zhiyuan Liu, Yong Li, Chenyu Zhu, Yunmao Huang, Xumeng Zhang

**Affiliations:** ^1^ College of Animal Science & Technology Zhongkai University of Agriculture and Engineering Guangzhou China

**Keywords:** apoptosis, chicken embryonic myogenic cells, lipopolysaccharide, polysaccharide of *Atractylodes macrocephala* Koidz, proliferation and differentiation

## Abstract

**Background:**

Lipopolysaccharide (LPS) can induce systemic inflammation and affect the growth and development of poultry. As a kind of traditional Chinese medicine, polysaccharide of *Atractylodes macrocephala* Koidz (PAMK) can effectively improve the growth performance of animals and improve the immunity of animal bodies.

**Objectives:**

The purpose of this study was to investigate the effects of PAMK on LPS‐induced inflammatory response, proliferation, differentiation and apoptosis of chicken embryonic myogenic cells.

**Methods:**

We used chicken embryonic myogenic cells as a model by detecting EdU/MYHC immunofluorescence, the expression of inflammation, proliferation, differentiation‐related genes and proteins and the number of apoptotic cells in the condition of adding LPS, PAMK, belnacasan (an inhibitor of Caspase1) or their combinations.

**Results:**

The results showed that LPS stimulation increased the expression of inflammatory factors, inhibited proliferation and differentiation, and excessive apoptosis in chicken embryonic myogenic cells, and PAMK alleviated these adverse effects induced by LPS. After the addition of belnacasan (inhibitor of *Caspase1*), apoptosis in myogenic cells was inhibited, and therefore, the number of apoptotic cells and the expression of pro‐apoptotic genes *Caspase1* and *Caspase3* were increased. In addition, belnacasan inhibited the increased expression of inflammatory factors, inhibited proliferation, differentiation and excessive apoptosis in chicken embryonic myogenic cells induced by LPS.

**Conclusions:**

This study provides a theoretical basis for further exploring the mechanism of action of PAMK and exogenous LPS on chicken embryonic myogenic cells and lays the foundation for the development and application of green feed additives in animal husbandry industry.

## Graphical abstract

This study showed that PAMK can alleviate the inflammatory response, proliferation inhibition, differentiation inhibition and excessive apoptosis of chicken embryonic myogenic cells induced by LPS. The inhibition of apoptosis in myogenic cells was also effective in alleviating the inflammatory response, proliferation inhibition, differentiation inhibition and excessive apoptosis induced by LPS in chicken embryonic myogenic cells. This study will provide a theoretical basis for future efforts to improve the growth performance of poultry by adding PAMK to feed.

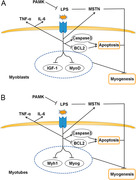

## 1 INTRODUCTION

As modern intensive farming develops rapidly, the barn environment greatly impacts livestock and poultry growth and health. The poultry body can normally resist infections like germs under normal circumstances. However, improper feeding practices can deteriorate barn environment and increase the reproduction of bacterial pathogens, which can cause a build‐up of lipopolysaccharide (LPS) in the environment, triggering a stress response in poultry, which can cause a series of inflammatory reactions (Demmers et al., 2003).

LPS, a component of gram‐negative bacteria, can cause varying inflammatory responses in poultry depending on how it is introduced (Kayagaki et al., 2013). When inflammation occurs, cells release chemicals that trigger inflammatory responses, such as cytokines, pro‐inflammatory factors and anti‐inflammatory factors. *TNFα* is a potent pro‐inflammatory and immunomodulatory factor (Xia et al., 2016), which has an important role in many diseases that promote inflammation‐related conditions, such as rheumatoid arthritis, Crohn's disease and ulcerative colitis (Batra et al., 2018). As a signalling cytokine, *IL6* is important for triggering acute phase responses, immune responses, haematopoiesis and inflammatory responses against pathogen invasions or tissue damage (Kishimoto, 2010), and inflammatory diseases can produce excessive amounts of *IL6* (Garbers et al., 2018). In broilers, Xie et al. (2000) discovered reduced body weight gain and a rise in blood *IL6* 24 h after LPS injection. Researchers found that broilers that developed inflammatory responses had slow growth and low production performances (Takahashi et al., 2000).

Vertebrate skeletal muscle develops from mesenchymal stem cells, where they proliferate and differentiate into mononuclear myogenic myoblasts, and multinucleated myotubes are formed when several myogenic myoblasts fuse and join, which then grow and differentiate into mature myofibres. Myofibre formation is regulated by complex molecular processes, and as skeletal muscle develops, a complex network of genes, signalling pathways and transcription factors must be regulated, and signal transduction pathways and regulatory mechanisms associated with these genes are relatively complex. Major escalating regulators involved in the regulation of myofibre growth and differentiation are the myogenic regulatory family (MRF) and myostatin (*MSTN*). *MyoD* and *Myog* are members of the MRF (Zammit, 2017), which are expressed when external stimuli activate satellite cells and promote hypertrophy of muscles (Dieli‐Conwright et al., 2009)*. Myog i*s a myogenic differentiation factor that activates muscle proteins and stimulates the formation of myofibres (Kim et al., 2009). Generally, *MSTN* is expressed specifically in muscle tissue and negatively regulates muscle growth, as well as influences fat metabolism and developmental processes. A member of the insulin growth factor family, *IGF1* influences muscle development by regulating skeletal muscle protein synthesis and has various physiological functions, including muscle proliferation (Lai et al., 2021)*. Myh1* gene polymorphisms are closely related to growth and sarcomere traits, the expression of distinct MyHC isoforms is a key determinant of contractility and metabolic properties of myofibres, and *Myh1* overexpression in muscle tissue may affect the type of myofibres (Ahn et al., 2018).

*TNFα* and *IL6* play a role in inducing inflammation in response to LPS through a Toll‐like receptor‐mediated signal transduction pathway. It has been reported that exogenous LPS can significantly reduce the diameter of C2C12 myotubes (Kayagaki et al., 2013). According to Yuko Ono et al., through the Toll4‐NF‐*κ*B signalling pathway, LPS inhibits the differentiation of C2C12 myoblasts by down‐regulating the expression of *Myog* and *MyoD* and increasing the expression of *MSTN*. In order for adult myoblast differentiation to occur, cell contact and proper apoptosis resulting from a certain density of adult myoblasts are required (Krauss et al., 2017; Yu & Baylies, 2013). *Bcl2* and *Caspase3* play significant regulatory roles during apoptosis (Dong et al., 2011; Park et al., 2009).

*Atractylodes macrocephala* is a traditional Chinese medicine, which plays roles in anti‐tumour, anti‐inflammatory and antioxidant processes. One of the main bioactive components of *A. macrocephala*, a plant commonly used as an immunomodulator, is polysaccharide of *A. macrocephala* Koidz (PAMK). In addition to its antioxidant properties and anti‐ageing properties, PAMK also acts on cardiomyocytes and is also clinically used for treating hepatitis and tumour disease. Researchers found that PAMK prevented the cyclophosphamide‐induced decrease of *IL1β*, *IL5*, *IL6* and *TGFβ* in thymus and lymphocytes of geese and deactivated the cyclophosphamide‐induced decrease in the activation level of T lymphocytes. Guo et al. (2021) found that PAMK was able to reduce inflammation and oxidative stress in mice induced by LPS and exerted protective effects at the early stage of LPS invasion in the liver. Xu et al. (2015) found that the PAMK significantly decreased the expression of *IL2*, *IL4*, *TNFα* and *IFNγ* in the spleen and thymus of chickens subjected to heat stress.

Belnacasan (VX‐765) is an orally bioactive prodrug of VRT‐043198 that inhibits *Caspase1* activity once metabolized by esterases in the body. The results of in vivo studies in mice showed that belnacasan not only inhibits *Caspase1* activity and suppresses the release of *IL1β* and IL18 (Mckenzie et al., 2018) but also inhibits *Caspase1*‐mediated scorching of renal tubular epithelial cells and inflammation ameliorating diabetic kidney injury.

Our previous study has demonstrated that LPS can enhance the immune function of animal organism, and LPS can induce inflammatory response and pro‐growth effect of PAMK in poultry, and LPS can promote the proliferation, differentiation and apoptosis of goose embryonic myogenic myoblasts (Wang et al., 2022). However, there are few studies of LPS in chicken embryonic myogenic cells. Therefore, in this study, we used chicken embryonic myogenic cells as a model to explore the effects of PAMK and LPS on proliferation, differentiation and apoptosis by detecting the expression of EdU and MYHC immunofluorescence; inflammation, proliferation, differentiation‐related genes and proteins and the number of apoptotic cells under the condition of adding belnacasan (an inhibitor of *Caspase1*), to investigate whether the effect of LPS alleviated by PAMK on the proliferation and differentiation of adult myoblasts was through the apoptotic pathway. This study will provide a theoretical basis for future efforts to improve the growth performance of poultry by adding PAMK to feed.

## 2 MATERIALS AND METHODS

### 2.1 Cell isolation and culturing

The leg muscle of chicken embryos at 12 days was transferred to a super clean platform, the big end of chicken embryo was opened with scissors, the chicken embryos were removed and transferred to a petri dish containing 2% penicillin streptomycin (Gibco) PBS solution by volume fraction, and the leg muscle was cleaned and collected. The collected leg muscle was transferred to a new petri dish containing 2% penicillin streptomycin PBS solution, and the skeletal muscle and fascia were carefully removed with scissors and tweezers: transferred to a 1.5 mL centrifuge tube containing 2% penicillin streptomycin PBS solution, and blood cells were removed by repeated cleaning. The cleaned muscle tissue was transferred to the 1.5 mL centrifuge tube, cut into mince with clean small scissors, and then crushed with rolling rod. A volume of 3 mL 0.25% trypsin (Gibco) by volume was added, digested at 37°C for 5 min, then removed and beaten, and the digestion time was increased according to the situation. DMEM‐F12 (Gibco) solution containing 20% foetal bovine serum (FBS, Gibco) by volume was added to terminate digestion, followed by 200 mesh cell sieve (Gibco), centrifugation at 4°C for 5 min at 1000 r/min, and supernatant was discarded. The cells were suspended by DMEM‐F12 solution with 20% FBS by volume. The cells were purified by differential adhesion purification method, and the cell suspension was inoculated into petri dish or culture flask and cultured at 39°Cand 5% CO_2_ for 1 h. After purification, the cells were transferred to DMEM‐F12 medium containing 20% FBS for culture. When the cell density reached 70%–80%, the cells were transferred to DMEM‐F12 medium containing 2% horse serum (HS, Sigma) for the induction of differentiation.

### 2.2 Cell treatments

In proliferation phase, drug treatment was performed when the cell density reached 70%–80%, and the cells were treated in eight groups: control group (PC group: cultured in normal proliferation medium), PAMK group (PP group: cultured in proliferation medium containing 15 µg/mL PAMK (Xi'an Tianyuan Biological Preparation Factory), LPS group (PL group: cultured in proliferation medium containing 500 ng/mL LPS [Serotype O55:B5]), LPS + PAMK group (PPL group: cultured in proliferation medium containing 500 ng/mL LPS and 15 µg/mL PAMK in proliferation medium), belnacasan group (PB group: 40 µM of belnacasan (MCE) in proliferation medium), belnacasan + PAMK group (PBP group: 40 µM of belnacasan and 15 µg/mL of PAMK in proliferation medium), belnacasan + LPS group (PPBL group: cultured in proliferation medium containing 40 µM of belnacasan and 500 ng/mL LPS), belnacasan + PAMK + LPS group (PBL group: cultured in proliferation medium containing 40 µM of belnacasan, 15 µg/mL PAMK and 500 ng/mL LPS).

In differentiation phase, drug treatment was performed when the cell fusion rate reached 70%–80%, and the cells were treated in eight groups: control group (DC group: cultured in normal differentiation medium), PAMK group (DP group: cultured in differentiation medium containing 15 µg/mL PAMK), LPS group (DL group: cultured in differentiation medium containing 500 ng/mL LPS), LPS + PAMK group (DPL group: cultured in differentiation medium containing 500 ng/mL LPS and 15 µg/mL PAMK in differentiation medium), belnacasan group (DB group: differentiation medium containing 40 µM of belnacasan), belnacasan + PAMK group (DBP group: differentiation medium containing 40 µM of belnacasan and 15 µg/mL of PAMK), belnacasan + LPS group (DBL group: cultured in differentiation medium containing 40 µM of belnacasan and 500 ng/mL LPS), belnacasan + PAMK + LPS group (DPBL group: cultured in differentiation medium containing 40 µM of belnacasan, 15 µg/mL PAMK and 500 ng/mL LPS).

### 2.3 EdU cell proliferation assay

After 24 h of cell treatment, myoblast proliferation assay was performed according to the instructions for use of the EdU kit (C10310‐3, RiboBio). The procedure was as follows (96‐well plate was used as an example): prepare EdU medium by diluting appropriate amount of proliferation medium and reagent A at a ratio of 1000:1. After the treated myogenic myoblasts were washed, 100 µL of EdU medium was added to each well, and the cells were incubated for 2 h. The EdU medium was discarded, and the cells were washed twice with PBS for 5 min each time to elute the EdU not infiltrated with DNA. Add 100 µL 4% paraformaldehyde to each well, incubate for 30 min at room temperature, discard the fixative, add 100 µL 2 mg/mL glycine solution, incubate for 5 min on a decolorized shaker and discard the glycine solution; add 100 µL PBS to each well, wash for 5 min on a decolorized shaker and discard the PBS. Take 9.38 mL deionized water, add 500 µL reagent B, 100 µL reagent C and 30 µL reagent D in order. A volume of 100 µL reagent C, 30 µL reagent D and 88 mg reagent E were added to the Apollo staining reaction solution. Add 100 µL Apollo staining reaction solution to each well, incubate in decolorization shaker at room temperature for 30 min and then discard the staining reaction solution; add 100 µL permeant (0.5% Triton X‐100 in PBS) to each well and incubate in decolorization shaker for 10 min, then discard the permeant and wash once with PBS for 5 min. 1× Hoechst 33342 reaction solution was prepared by diluting deionized water and reagent F at a ratio of 100:1. Add 100 µL 1× Hoechst 33342 reaction solution to each well, incubate in a decolorized shaker for 30 min at room temperature and then discard the staining reaction solution; wash with PBS 1–3 times for 5 min each time. Use an inverted fluorescence microscope (IX73P1F, Olympus) for image acquisition and analysis and use Image‐Pro Plus 6.0 for cell EdU positive rate statistics. Staining results: Nuclei were blue and nuclei of EdU‐positive cells were green.

### 2.4 Real‐time quantitative PCR

Cells were harvested at 0.5, 3 and 24 h of cell treatment, respectively, and the collected cells were extracted with RNA by Trizol method and reverse transcribed into cDNA using Reverse Transcription Kit (FSQ‐301, TOYOBO), and real‐time quantitative PCR was performed on Light Cycler 480 II (QuantStudio 7 Flex, Thermo Fisher Scientific) with the following reaction procedure. By comparing with GenBank in the NCBI database, real‐time PCR primers were designed by software Primer Premier 5.0 (Table 1). Pre‐denaturation 95°C for 10 min; denaturation 95°C for 5 s, annealing 60°C for 1 min, extension 72°C for 30 s, 40 cycles. The inflammation‐related genes *IL6*, *TNFα*, muscle‐specific expression genes *MyoD*, *MSTN*, *Myh1*, *Myog* and *IGF‐1* and apoptosis‐related genes *Bcl2* and *Caspase3* were detected and analysed using GAPDH as an internal reference gene. The testing time was 0.5 and 3 h for *IL6* and *TNFα* genes and 24 h for the other genes.

**TABLE 1:** Primer sequences for RT‐qPCR.

Gene name	Forward (3′ ‐5′)	Reverse (3′‐5′)
*GAPDH*	TCTGTCGTGGACCTGACCTGC	GCCAGCACCCGCATCAAA
*IL1β*	AAGTGAGGCTCAACATTGCG	CGGTAGAAGATGAAGCGGGT
*IL6*	TTCGACGAGGAGAAATGCTT	CCTTATCGTCGTTGCCAGAT
*TNFα*	ATGAACCCTCCTCCGTACAC	AGAGGCCACCACATGATAGC
*MyoD*	AAGGCGTGCAAGAGGAAGAC	TGGTTGGGGTTGGTGGA
*IGF1*	AGGTCGTCCATCGTAGTCCTTGCA CTTTTAAGAAGCAATGGA	ACAGCGTCGTTATCGTTCCTGCAAACACAGGCCAAGGTAG
*Myh1*	CTCCTCACGCTTTGGTAAAT	GCTCTGGCTTCTTGTTGGAC
*Myog*	CCCGAGCACTGCCCCGGGCAAT	CCCGAGCACTGCCCCGGGCAAT
*Caspase1*	ATACACTTGCCACGGGAGACAC	ACCAGGAAGGTGCTGTCAGAGG
*Caspase3*	TATTCTACTGCTCCAGGCTAT	CAAGTTTCCGTGCATTTT
*Bcl2*	TCCTCTCTCCCTTCCTCTTGCT	CCGGTTATCGTAGCCTCTTCTC

### 2.5 Western blot

Cells were harvested at 0.5 and 3 h of cell treatment to detect the expression levels of IL6 (ab9324, 1:5000 dilution, Abcam) and TNFα (WL01581, 1:1000 dilution, Wanlei) proteins; cells were harvested at 24 h of cell treatment to detect the expression levels of MyoD (ab16148, 1:10000 dilution, Abcam), MYHC (M4276, 1:10000 dilution, Sigma) and MSTN (ab9324, 1:1000 dilution, Abcam) proteins, with GAPDH (ab181602, 1:10000 dilution, Abcam) selected as the internal reference protein. The collected cells were lysed on ice with RIPA buffer, EDTA and PMSF. The cell lysate was centrifuged at 12,000 × *g* for 5 min and the supernatant was transferred to a new EP tube, and the protein concentration of each sample was determined using the BCA protein assay kit. Equal amounts of protein samples to be measured were subjected to SDS–PAGE electrophoresis, set at a constant pressure of 80 V for 15 min (for concentrated gels) and 100 V for 60 min (for separated gels). After electrophoresis, the gels were removed and placed in a transfer apparatus in the order of bottom‐up filter paper‐PVDF membrane‐gel‐filter paper, set at a constant current of 200 A. The transfer time depended on the protein size, and the proteins were transferred to PVDF membranes, which were subsequently closed with 5% skim milk at room temperature for 1–2 h. After closure, the membranes were placed in antibody incubation boxes, and primary antibody was added and incubated overnight at 4°C on a horizontal shaker. Incubation: After incubation with primary antibody, the PVDF membrane is placed in 1 × TBST and washed three times for 5 min each time on a decolorized shaker, and the secondary antibody (HRP labelled goat anti‐mouse IgG, ab181602, 1:10,000 dilution, Abcam; or HRP labelled goat anti‐rabbit IgG, WLA023a, 1:10000 dilution, Wanlei) is incubated in the same way for 1 h at room temperature on a shaker. The images were analysed with Tanon 5200 Multi device (Tanon).

### 2.6 Fluorescence activated cell sorting

After 24 h of cell treatment, the walled cells were trypsinized again, and the terminated digested cells were subjected to induction of apoptosis according to the instructions of Apoptosis Detection Kit (E‐CK‐A211, Elabscience). The cells were collected by centrifugation at 1000 rpm for 5 min, the supernatant was discarded, and the cells were washed twice with pre‐chilled PBS to adjust the number of cells per tube to 0.2–1.0 × 10^6^. Add 400 µL 1× binding buffer to resuspend the cells. Add 5 µL FITC‐Annexin V to each tube and react at room temperature for 15 min; then add 10 µL PI and mix lightly for 5 min at 4°C. After the reaction, complete the assay in BD Accuri C6 device (BD) within 1 h. The appropriate channels were selected, and additional blank tubes (without fluorescent reagent) and PI single‐stained tubes (with PI reagent only) were set up for voltage adjustment of the flow instrument and compensation of fluorescent channels, and the results were analysed by FlowJo 7.6 software.

### 2.7 Statistical analysis

All statistical analyses were performed using SPSS 24.0, and graphs were made with GraphPad Prism 7.0. Two‐factor ANOVA was used to calculate differences between groups, and relative quantitative analysis was performed using the 2^−△△^ *^Ct^* method, with *p *< 0.01 indicating a highly significant difference; *p *< 0.05 indicating a significant difference; and *p *> 0.05 indicating a non‐significant difference.

## 3 RESULTS

### 3.1 Effects of PAMK and exogenous LPS on the expression of inflammatory factors in apoptosis‐suppressed chicken embryonic myogenic cells

**FIGURE 1:**
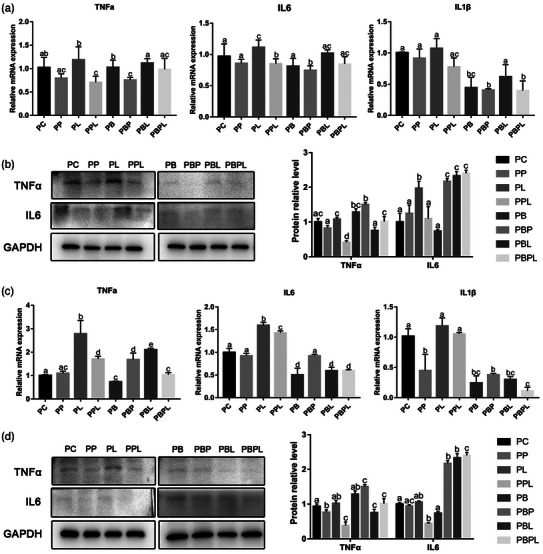
Gene and protein expression results of polysaccharide of *Atractylodes macrocephala* Koidz (PAMK), exogenous lipopolysaccharide (LPS) and belnacasan‐treated myoblasts at 0.5 and 3 h during proliferation phase: (a) Relative expression level of *TNFα, IL6*, *IL1β* mRNA at 0.5 h during proliferation phase; (b) Western blot picture and quantitative results of TNFα and IL6 at 0.5 h during proliferation phase; (c) relative expression level of *TNFα, IL6*, *IL1β* mRNA at 3 h during proliferation phase; (d) Western blot picture and quantitative results of TNFα and IL6 at 3 h during proliferation phase. Different letters marked in the graphs indicate significant differences (*p *< 0.05), and the same letters marked indicate insignificant differences (*p* > 0.05).

The expression of inflammatory factors in myoblasts during proliferation phase was detected. Compared with the PC group, the mRNA and protein expression level of *TNFα*, *IL6* and *IL1β* decreased after 0.5 and 3 h of PAMK treatment but did not reach significance (*p* > 0.05). After 0.5 and 3 h of LPS treatment, the mRNA and protein expression level of *TNFα*, *IL6* and *IL1β* increased, and the mRNA expression level of *TNFα* and *IL6* reached a significant difference at 3 h (*p *< 0.05). After PAMK + LPS treatment for 0.5 and 3 h, the mRNA and protein expression of *TNFα*, *IL6* and *IL1β* decreased compared with LPS group, and the mRNA and protein expression of *TNFα* and *IL6* reached a significant difference at 0.5 and 3 h (*p *< 0.05). Compared with PC group, the mRNA expression of *TNFα* decreased in 0.5 h PPB group and PBPL group, slightly increased in PBL group and significantly increased in 3 h PBP group and PPL group (*p *< 0.05). The mRNA expression of *IL1β* decreased after belnacasan treatment for 0.5 and 3 h, and the decrease level was higher in the PBPL group than in the PBL group. Except that the mRNA expression of *IL6* in the 0.5 h PBL group was not significantly different from that in the PC group, the expression levels of other groups were decreased after 0.5 and 3 h of belnacasan treatment, and the decrease level in the PBPL group was higher than that in the PBL group. However, the mRNA and protein expression levels of TNFα and IL6 were higher in the treatment group with belnacasan than in the treatment group without belnacasan (*p *< 0.05) (Figure 1a–d).

**FIGURE 2:**
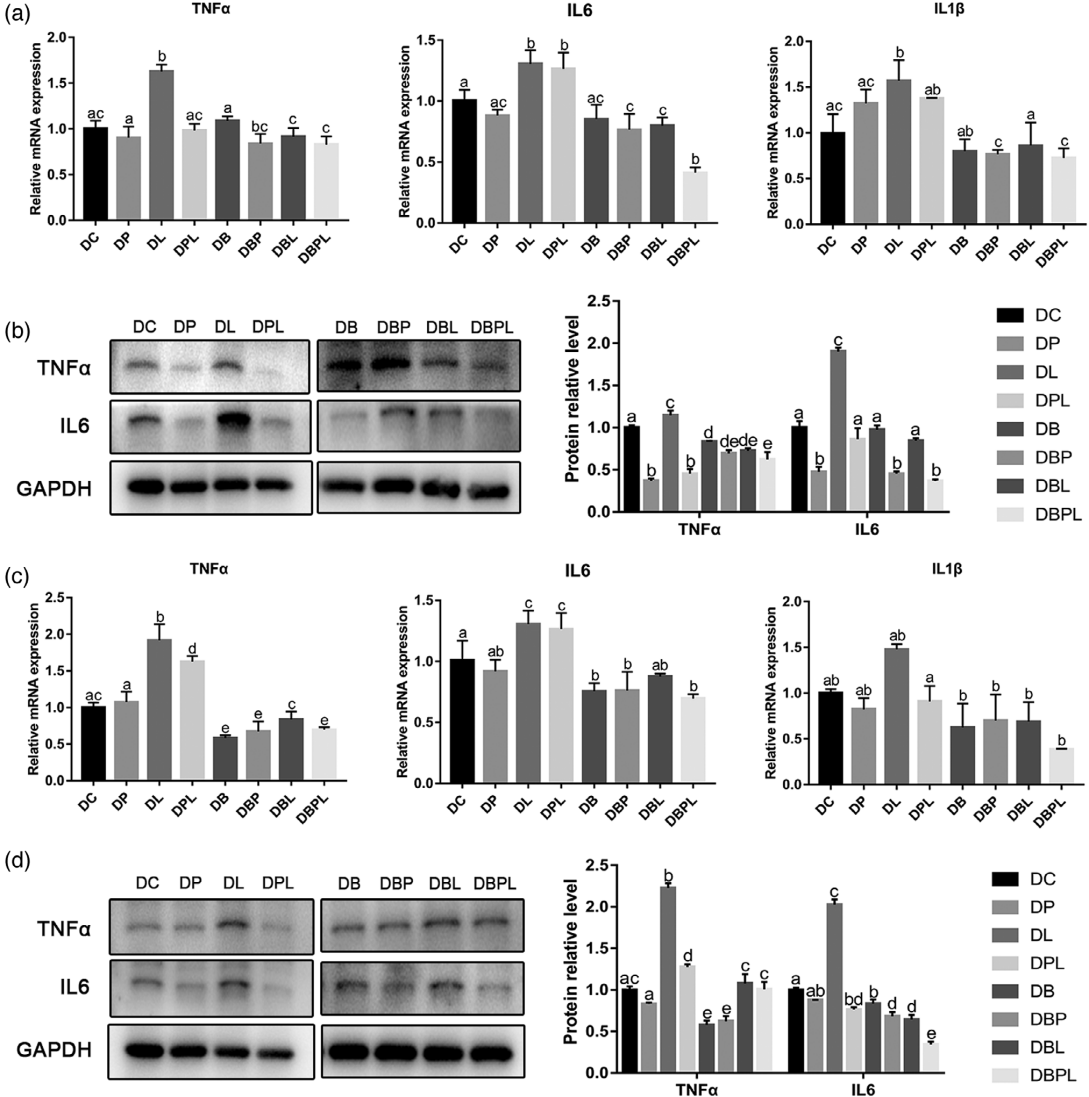
Gene and protein expression results of polysaccharide of *Atractylodes macrocephala* Koidz (PAMK), exogenous lipopolysaccharide (LPS) and belnacasan‐treated myotubes at 0.5 and 3 h during differentiation phase: (a) Relative expression level of *TNFα, IL6*, *IL1β* mRNA at 0.5 h during differentiation phase; (b) Western blot picture and quantitative results of TNFα and IL6 at 0.5 h during differentiation phase; (c) Relative expression level of *TNFα, IL6*, *IL1β* mRNA at 3 h during differentiation phase; (d) Western blot picture and quantitative results of TNFα and IL6 at 3 h during differentiation phase. Different letters marked in the graphs indicate significant differences (*p *< 0.05), and the same letters marked indicate insignifazicant differences (*p* > 0.05).

The expression of inflammatory factors in myotubes during differentiation phase was detected. Compared with the DC group, the mRNA and protein expression level of *TNFα*, *IL6* and *IL1β* decreased after PAMK treatment for 0.5 and 3 h, which was consistent with the expression trend in the proliferation phase, and the protein expression level reached significance at 0.5 h (*p* > 0.05). After LPS treatment for 0.5 and 3 h, the mRNA and protein expression level of *TNFα*, *IL6* and *IL1β* increased significantly (*p *< 0.05). After PAMK + LPS treatment for 0.5 and 3 h, the mRNA expression of *TNFα*, *IL6* and *IL1β* decreased significantly compared with DL group (*p *< 0.05). Compared with the DC group, the expression of *TNFα* mRNA in the 0.5 h DB group was higher than that in the DC group, and the expression levels of other groups were decreased after 0.5 and 3 h treatment with belnacasan. The mRNA expression level of *IL6* and *IL1β* also showed a downward trend after belnacasan treatment for 0.5 and 3 h, and the decrease level was higher in the DBPL group than in the DBL group. In addition, TNFα and IL6 protein expression levels were significantly lower in the treated group with the addition of belnacasan compared to the treated group without the addition of belnacasan (*p *< 0.05) (Figure 2a–d). The above results showed that PAMK could alleviate the increase of inflammatory factor expression in chicken embryonic myogenic cells induced by LPS.

### 3.2 Effects of PAMK and exogenous LPS on the proliferation of apoptosis‐suppressed chicken embryonic myogenic cells

**FIGURE 3:**
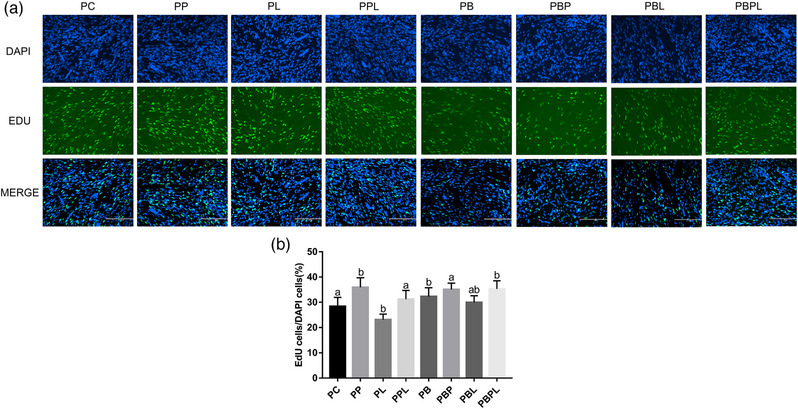
EdU results of polysaccharide of *Atractylodes macrocephala* Koidz (PAMK), exogenous lipopolysaccharide (LPS) and belnacasan‐treated myoblasts for 24 h during proliferation phase: (a) EdU staining results after 24 h treatment during proliferation phase; (b) EdU quantification results (200×, scale bar = 200 µm). Different letters marked in the graphs indicate significant differences (*p *< 0.05), and the same letters marked indicate insignificant differences (*p* > 0.05).

The results of EdU staining showed that the EdU positive rate of myogenic cells treated with PAMK for 24 h was significantly higher (*p *< 0.05) compared with the PC group, and the EdU positive rate of cells in the LPS‐treated group was significantly lower (*p *< 0.05) compared with the PL group; the EdU positive rate of myoblasts in the PPL‐treated group again tended to be significantly higher (*p *< 0.05). The EdU‐positive rate of myogenic cells was significantly higher in all treatment groups with belnacasan compared with the PC group (*p *< 0.05), with the PBL‐treated group having the lowest EdU‐positive rate among all treatment groups. There was a trend of significantly higher EdU positivity in all treated groups of myoblasts with belnacasan addition compared to the PC group (Figure 3a,b).

**FIGURE 4:**
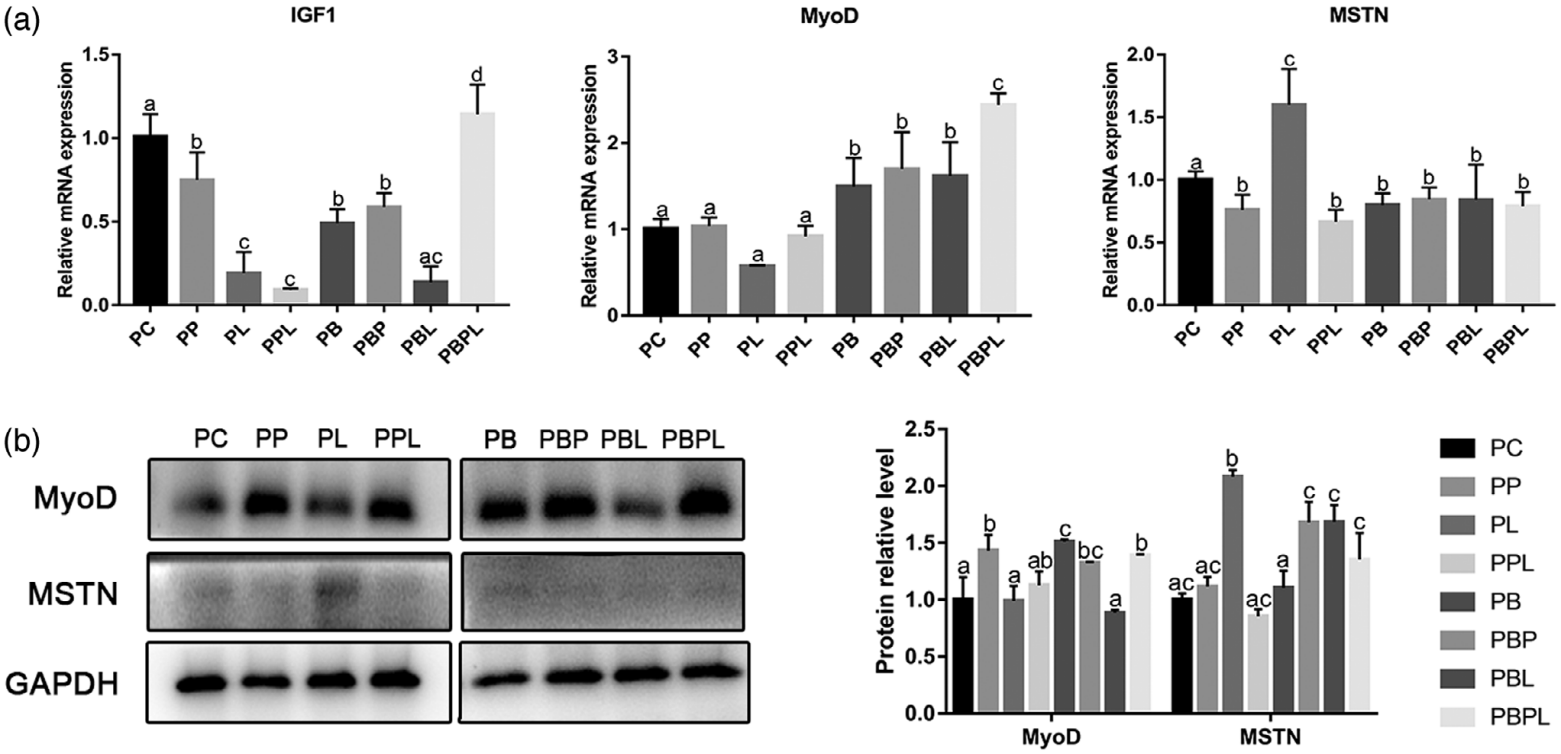
Gene and protein expression results of polysaccharide of *Atractylodes macrocephala* Koidz (PAMK), exogenous lipopolysaccharide (LPS) and belnacasan‐treated myoblasts for 24 h during proliferation phase: (a) Relative expression level of *IGF1, MyoD* and myostatin *(MSTN)* mRNA; (b) Western blot picture and quantitative results of MyoD and MSTN. Different letters marked in the graph indicate significant differences (*p *< 0.05), and the same letters marked indicate non‐significant differences (*p* > 0.05).

The expression level of myogenic proliferation‐related genes showed that after 24 h of PAMK and LPS stimulation in myogenic cells, the expression level of *IGF1* decreased significantly (*p *< 0.05) in all treatment groups compared with the PC group, with a more significant decrease in the PL and PPL groups. The expression levels of *MyoD* were not significantly different in the PP group compared with the PC group, whereas the expression levels decreased significantly in the PL group (*p *< 0.05), and significantly increased in the PPL group compared with the PL group (*p *< 0.05). Although the expression levels of myogenic inhibitor *MSTN* were significantly lower in the PP group and higher in the PL group than in the PC group, the expression levels in the PPL group were significantly lower than in the PL group (*p *< 0.05). After 24 h of myogenic cells stimulation by belnacasan, PAMK and LPS, the expression level of *IGF1* was significantly decreased in PB, PBP and PBL groups compared with PC group (*p *< 0.05), and the expression level of *IGF1* was significantly increased in PBPL group compared with other treatment groups and even PC group (*p *< 0.05). The protein expression levels of MyoD and MSTN were consistent with those of mRNA in the treatment groups without the addition of belnacasan. The mRNA and protein expression level of *MyoD* was significantly increased compared with PC group and compared to the PC group and other treatment groups (*p *< 0.05). In contrast, the mRNA expression level of myogenic inhibitor *MSTN* was lower than that of the PC group in all treatment groups but did not reach a significant difference (*p* > 0.05), but the protein expression level of MSTN was significantly higher in the treatment group with belnacasan than in the treatment group without belnacasan (*p *< 0.05) (Figure 4a,b). The above results indicated that PAMK could alleviate the LPS‐induced proliferation inhibition of chicken embryonic myogenic cells.

### 3.3 Effects of PAMK and exogenous LPS on the differentiation of apoptosis‐suppressed chicken embryonic myogenic cells

**FIGURE 5:**
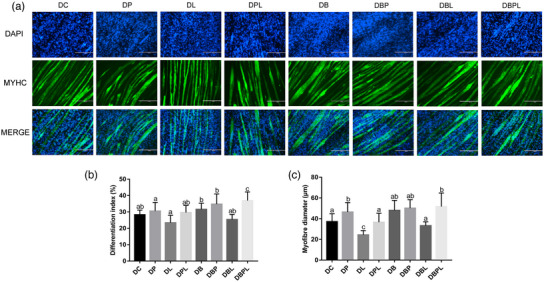
MYHC immunofluorescence results of polysaccharide of *Atractylodes macrocephala* Koidz (PAMK), exogenous lipopolysaccharide (LPS) and belnacasan‐treated myotubes for 24 h during differentiation phase: (a) MYHC immunofluorescence staining results after 24 h treatment with PAMK, exogenous LPS and belnacasan during differentiation stage; (b) MYHC immunofluorescence myotube fusion rate quantification results; (c) MYHC immunofluorescence myotube diameter quantification results (200×, scale bar = 200 µm). Different letters marked in the graphs indicate significant differences (*p *< 0.05), and the same letters marked indicate insignificant differences (*p* > 0.05).

The results of MYHC immunofluorescence staining showed that the differentiation index and myofibre diameter treated with PAMK for 24 h were significantly higher (*p *< 0.05) than those of the DC group, whereas the differentiation index and myofibre diameter in the PL group were significantly lower (*p *< 0.05); compared with those in the DL group, the differentiation index and myofibre diameter in the DPL group were significantly higher (*p *< 0.05). After 24 h of belnacasan treatment, differentiation index and myofibre diameter were significantly increased (*p *< 0.05) in all the treated groups, except for the DBL group, where differentiation index and myofibre diameter decreased compared with the PC group (Figure 5a–c).

**FIGURE 6:**
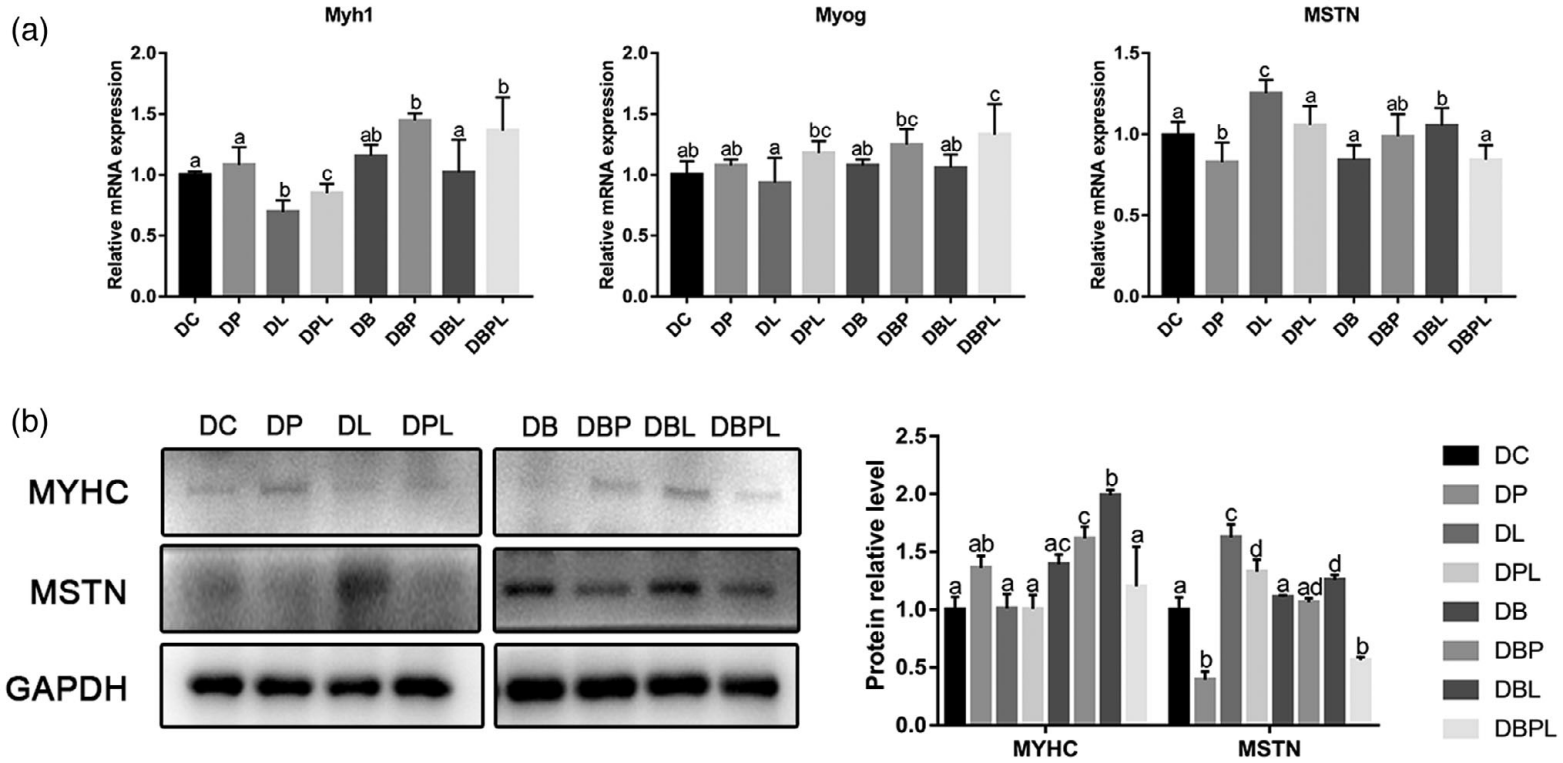
Gene and protein expression results of polysaccharide of *Atractylodes macrocephala* Koidz (PAMK), exogenous lipopolysaccharide (LPS) and belnacasan treated for 24 h during differentiation phase: (a) Relative expression level of *Myh1, Myog* and myostatin (*MSTN)* mRNA; (b) Western blot picture and quantitative results of MYHC and MSTN. Different letters marked in the graphs indicate significant differences (*p *< 0.05), and the same letters marked indicate insignificant differences (*p* > 0.05).

The expression level of genes related to myogenic differentiation showed that after 24 h of PAMK stimulation in myotubes, the expression level of *Myh1* and *Myog* increased but did not reach a significant difference (*p* > 0.05) and the expression level of *MSTN* decreased significantly (*p *< 0.05) compared to the DC group. The expression level of *Myh1* and *Myog* was decreased significantly (*p *< 0.05) in the DL group, and the expression level of *MSTN* expression levels was significantly increased in the DL and DPL groups (*p *< 0.05). The expression level of *Myh1* and *Myog* was significantly increased in the DPL group compared with the DL group (*p *< 0.05), and the expression level of *MSTN* was significantly decreased in the DBL group (*p *< 0.05). The expression level of *Myh1* was increased in the DB, DBP and DBPL groups compared with the DC group, whereas there was no significant difference between the DBL and PC groups (*p* > 0.05). The expression levels of *Myog* were increased compared with the DC group. The expression level of MSTN in the DBP group was marginally higher than that in the DC group, but that of the DB and DBPL groups was lower than the DC group (*p* > 0.05). Compared with the DC group, the protein expression level of MYHC increased in the DP group and decreased in the DL group, and the protein expression trend of MSTN was the opposite. The protein expression level of MYHC was higher in the treatment group with belnacasan than in the treatment group without belnacasan, and the expression level of MSTN was lower in the treatment group without belnacasan (Figure 6a,b). The above results indicated that PAMK could alleviate the LPS‐induced inhibition of differentiation in chicken embryonic myogenic cells.

### 3.4 Effects of PAMK and exogenous LPS on apoptosis of apoptosis‐suppressed chicken embryonic myogenic cells

**FIGURE 7:**
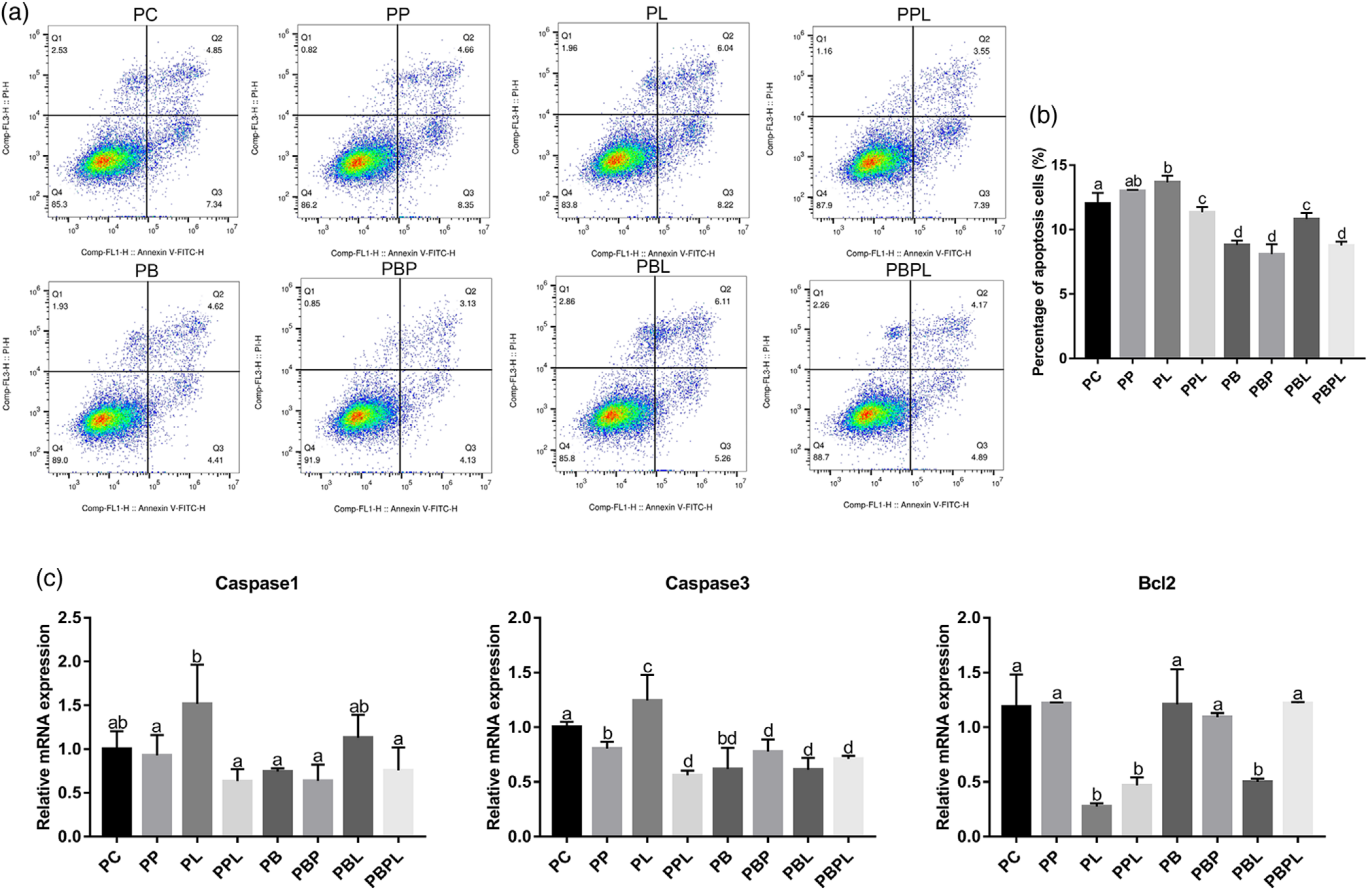
FACS and apoptotic gene expression results of polysaccharide of *Atractylodes macrocephala* Koidz (PAMK), exogenous lipopolysaccharide (LPS) and belnacasan‐treated myoblasts for 24 h during proliferation phase: (a) FACS results of PAMK, exogenous LPS and belnacasan‐treated myoblasts for 24 h during proliferation phase; (b) quantitative results of FACS in proliferation phase; (c) expression level of apoptosis‐related genes. Different letters marked in the graphs indicate significant differences (*p *< 0.05), and the same letters marked indicate insignificant differences (*p* > 0.05).

The proliferation phase FACS results and apoptosis‐related gene expression level showed that the number of apoptotic cells increased slightly, the expression level of *Caspase1* and *Caspase3* decreased significantly (*p *< 0.05), and the expression level of *Bcl2* did not change significantly (*p* > 0.05) in myoblasts treated with PAMK for 24 h compared with the PC group. The number of apoptotic cells increased significantly, and the expression level of *Caspase1* and *Caspase3* increased significantly (*p *< 0.05) in the PL group. The expression level of *Caspase1* and *Caspase3* also increased significantly (*p *< 0.05), and the expression level of *Bcl2* decreased significantly (*p *< 0.05). Compared with the PL group, the number of apoptotic cells was significantly reduced in the PPL group, the expression levels of *Caspase1* and *Caspase3* were also significantly decreased, and the expression level group of *Bcl2* was significantly increased (*p *< 0.05). The expression level of the pro‐apoptotic gene *Caspase3* was significantly decreased in all treatment groups (*p *< 0.05), whereas the expression level of *Bcl2* was significantly decreased only in the PBL group (*p *< 0.05), and the differences between the other treatment groups and the control group were not significant (Figure 7a–c).

**FIGURE 8:**
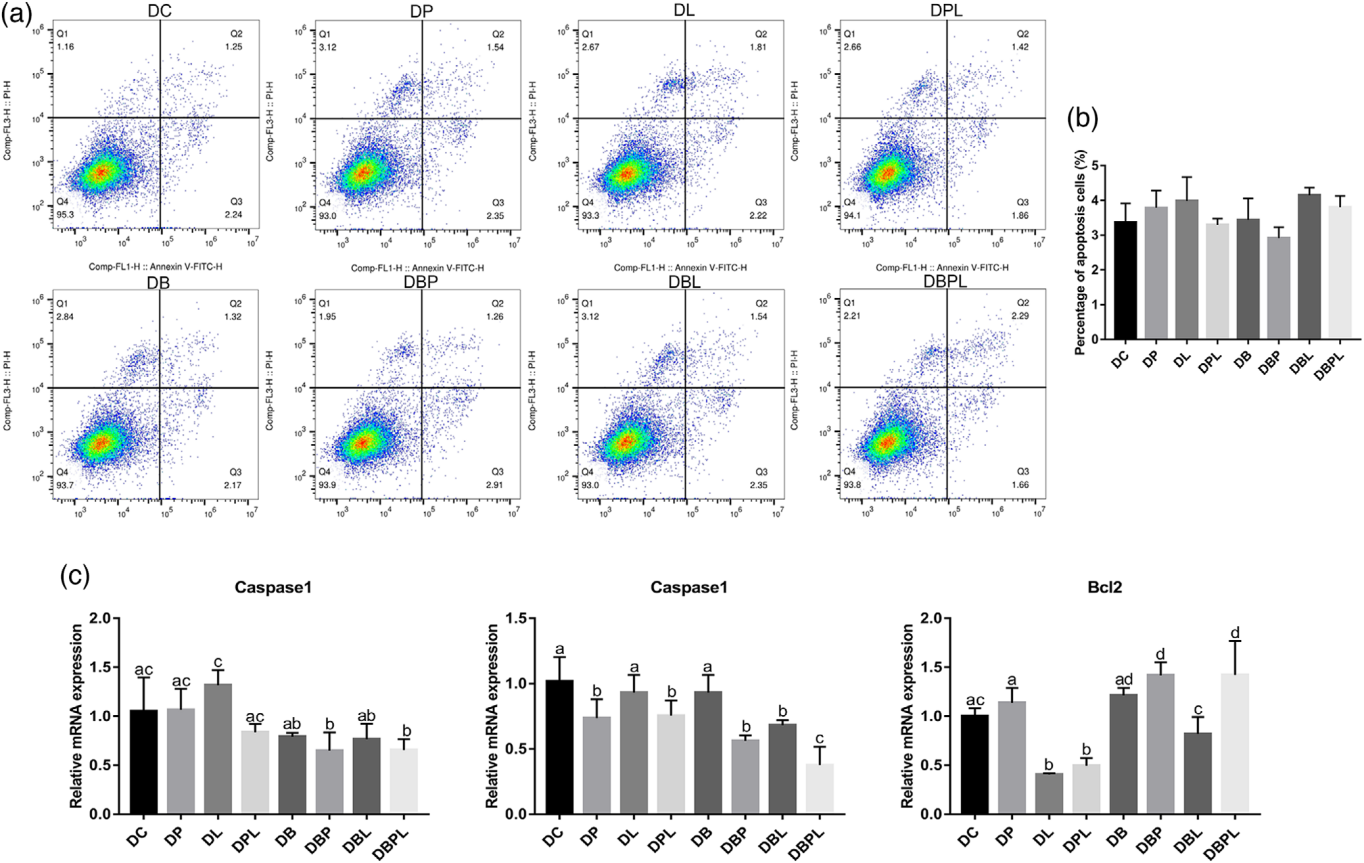
FACS and apoptotic gene expression results of polysaccharide of *Atractylodes macrocephala* Koidz (PAMK), exogenous lipopolysaccharide (LPS) and belnacasan‐treated myotubes at differentiation phase for 24 h: (a) FACS results of PAMK, exogenous LPS and belnacasan‐treated myotubes at differentiation stage for 24 h; (b) quantitative results of FACS in differentiation phase; (c) expression level of apoptosis‐related genes. Different letters marked in the graphs indicate significant differences (*p *< 0.05), and the same letters marked indicate insignificant differences (*p* > 0.05).

FACS results and apoptosis‐related gene expression level in the differentiation phase showed that, similar to the proliferation phase, the changes in the number of apoptotic cells in the differentiation phase without belnacasan treatment also showed a trend of increasing first and then decreased but did not reach a significant difference (*p* > 0.05). The number of apoptotic cells in the DBP group decreased compared with the control group, and the number of apoptotic cells in the DBL and DBPL groups was higher than that in the DC group, but the number of apoptotic cells in DBPL group was lower than that in DBL group (*p* > 0.05) (Figure 8a,b). The expression level of *Caspase1* and *Caspase3* decreased significantly in the DP group (*p *< 0.05), increased significantly in the DL group, decreased significantly in the DPL group compared to the DL group and decreased to a lower level than the control group; on the other hand, *Bcl2* had the opposite trend to the expression level of pro‐apoptotic genes (*p *< 0.05). The expression level of *Caspase1* and *Caspase3* decreased in all belnacasan‐treated groups, where the expression levels of *Caspase1* and *Caspase3* were also lower in the DBPL group than in the DBL group, whereas *Bcl2* was higher in all belnacasan‐treated groups than in the control group, except for the DBL group, which was lower than the control group (Figure 8c). These results indicated that PAMK could alleviate LPS‐induced excessive apoptosis in chicken embryonic myogenic cells.

## 4 DISCUSSION

There are many threats of bacterial diseases in poultry farming, and bacterial infections can cause oxidative stress and inflammatory responses in chickens, which negatively affect their weight gain and severely impact their health and productivity (Stanley et al., 2014). LPS is the main active component of the endotoxin produced by gram‐negative bacteria and is widely used to construct models of inflammation (Li et al., 2021). Although chickens are more tolerant to LPS than mammals, many studies have also found that a similar response to mammals occurs on chickens at certain doses of LPS infection (Konieczka et al., 2022; Oh et al., 2019; Zhang et al., 2021).

Most chronic inflammatory diseases affect skeletal muscle growth and development (Huang & Kraus, 2016). In this study, changes in the expression levels of inflammatory genes and proteins were detected after LPS stimulation of chicken embryonic myogenic cells. The results showed that the mRNA expression of *TNFα*, *IL6* and *IL1β* increased after LPS treatment in chicken embryonic myogenic cells for 0.5 and 3 h during proliferation and differentiation phases. This indicates that LPS can indeed cause an inflammatory response in chicken embryonic myogenic cells, and it also indicates that this experiment was successful in establishing a model of inflammatory damage in chicken embryonic myogenic cells.

The addition of PAMK reduced the expression level of inflammatory factors, and the expression level of inflammatory factors was lower with the addition of both PAMK and LPS than with the addition of LPS only. The expression levels of inflammatory factors *TNFα*, *IL1β* and *IL6* were also decreased to some extent after belnacasan treatment on chicken embryonic myogenic cells, and the expression level of *IL1β* was most significantly decreased compared with the control group. LPS is a major factor in bacterial pathogenesis and can cause a series of inflammatory responses in the body, including the release of inflammatory factors such as *TNFα*, *IL1β* and *IL6*, causing a variety of inflammatory symptoms such as metabolic disorders, fever and circulatory disturbances (Bode et al., 2012). The release of various pro‐inflammatory factors can attract neutrophils to the liver and release cytotoxic mediators such as proteases and reactive oxygen species, which in turn can cause indirect damage to the liver (Zhang et al., 2022). In addition, it was also found that LPS stimulated increased *TNFα* expression in C2C12 cells via TLR4‐NF‐*κ*B signalling pathway (Ono & Sakamoto, 2017). However, LPS stimulated *TNFα*, *IL1β* and *IL6* expression in goose embryonic myogenic myoblasts, and apoptosis inhibition by belnacasan cannot alleviate LPS‐induced inflammation in myogenic myoblasts (Wang et al., 2022), which may be due to the excessive concentration of added LPS and excessive production of inflammatory factors by the myoblasts. These studies are similar to the findings of the present experiment and can confirm that PAMK can alleviate LPS‐induced inflammatory responses in chick embryonic myogenic cells. Artemisia annua and its novel commercial product have also been proved to show remarkable capability to minimize *Clostridium perfringens* colonization in broiler intestines, modulation of mucus production, gut health integrity, immune organs and immune response (El‐Demerdash et al., 2023). Previous study also found the potential effects of Quercetin‐Loaded Nanoliposomes on Amoxicillin/Clavulanate‐induced Hepatic Damage by targeting the SIRT1/Nrf2/NF‐*κ*B signalling pathway and microbiota modulation (Abd El‐Emam et al., 2023). These studies will provide theoretical basis for future efforts to improve the growth performance of poultry.

In this study, we examined the proliferation and differentiation ability of chicken embryonic myogenic cells. The results showed that the EdU positive rate, myotube fusion rate and myotube diameter of myogenic cells treated with LPS were lower than those of the control group, whereas the EdU positive rate, myotube fusion rate and myotube diameter of myogenic cells treated with both PAMK and LPS were also lower than those of the LPS group; the EdU positive level increased after the addition of belnacasan. The expression level of *MyoD* and MYHC was also increased compared with the control group, whereas the expression level of *MSTN* was decreased. After the addition of belnacasan, the myotube fusion rate and myofibre diameter of differentiated myotubes increased in all treatment groups except the DBL group, and the expression level of *Myh1* and *MyoD*, genes related to myogenic differentiation, also increased compared with the control group. This indicates that PAMK can alleviate the LPS‐induced inhibition of proliferation and differentiation of chicken embryonic myogenic cells. Similar to the results of the present study, the reduction of PPARγ gene expression activity in broiler bursa of broilers inoculated with LPS resulted in the inhibition of B lymphocyte differentiation, development and proliferation in bursa. It has also been shown that LPS induces the muscle atrophy gene MuRF1 transcription in C2C12 myoblasts by regulating the AKT/FOXO1 signalling pathway, thereby inhibiting the differentiation of C2C12 myoblasts.

Belnacasan is a potent bioavailable non‐toxic small molecule inhibitor and a safe, effective and feasible drug. Therefore, the results found that belnacasan can reduce the number of apoptotic cells in chicken embryonic myogenic cells to a certain extent, also inhibit the expression of both *Caspase1* and *Caspase3* and promote the expression of *Bcl2*. In addition, LPS has been found to cause apoptosis and necrosis in hepatocytes (Jing et al., 2014). LPS can also induce apoptosis and mitochondrial dysfunction in C2C12 cells (Lim et al., 2022). The above study is similar to this paper, indicating that LPS promotes apoptosis. Our previous study also found that LPS promoted apoptosis in goose embryonic myogenic cells, but unlike the present study, LPS also promoted proliferation of goose embryonic myogenic cells (Wang et al., 2022), probably because proper apoptosis can promote cell proliferation to some extent. Moreover, belnacasan alleviated LPS‐induced inhibition of myogenic cell proliferation but failed to restore it to control level, whereas it was not significant in alleviating LPS‐induced inhibition of chicken embryonic myogenic cell differentiation. Belnacasan also improves mitochondrial function and myocardial contractility by inhibiting excessive activation of myocardial NLRP3 inflammatory vesicles after AMI, which could be a potential therapeutic tool. In this study, we examined apoptosis in chicken embryonic myogenic cells and found that LPS can reduce the number of apoptotic cells while promoting the expression of *Caspase1* and *Caspase3*, inhibiting the expression of *Bcl2*, which was alleviated by PAMK. To reduce apoptosis caused by LPS, this study added belnacasan, an inhibitor of *Caspase1*. However, the number of apoptotic cells increased slightly in PAMK‐treated adult myoblasts, probably because proper apoptosis is a prerequisite and necessary condition for myoblast differentiation (Yu et al., 2013), but excessive apoptosis would result in too few adult myoblasts to reach a state of cell‐to‐cell contact and the differentiation process would be inhibited. The results of this study suggest that PAMK may reduce LPS‐induced excessive apoptosis in chicken embryonic myogenic cells by reducing the number of apoptotic cells, downregulating *Caspase1* and *Caspase3*, and up‐regulating *Bcl2*. However, there are still few studies concerning LPS, belnacasan and PAMK on chicken embryonic myogenic cells, so the specific mechanism needs to be further investigated.

## 5 CONCLUSION

**FIGURE 9:**
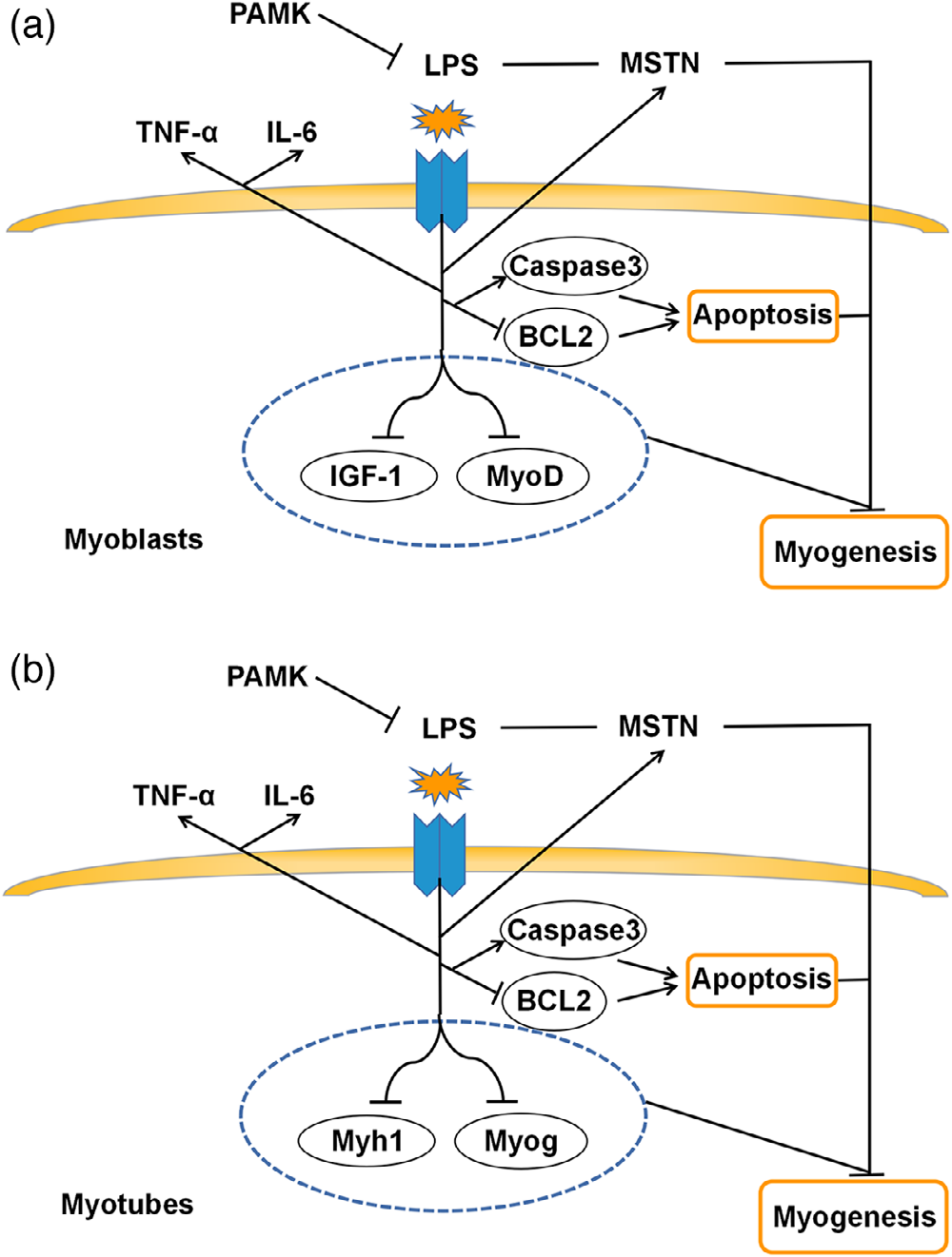
Mechanism of the influence of polysaccharide of *Atractylodes macrocephala* Koidz (PAMK) and exogenous lipopolysaccharide (LPS) on proliferation, differentiation and apoptosis in chicken embryonic myogenic cells: (a) mechanism of the influence of PAMK and exogenous LPS on proliferation and apoptosis in chicken embryonic myogenic cells; (b) mechanism of the influence of PAMK and exogenous LPS on differentiation and apoptosis in chicken embryonic myogenic cells.

In this study, we used a combination of PAMK, LPS and apoptosis‐inhibiting compounds to explore the effects of PAMK and LPS on the proliferation, differentiation and apoptosis of chicken embryonic myogenic cells. The results showed that PAMK can alleviate the inflammatory response, proliferation inhibition, differentiation inhibition and excessive apoptosis of chicken embryonic myogenic cells induced by LPS. In addition, the inhibition of apoptosis in myogenic cells was also effective in alleviating the inflammatory response, proliferation inhibition, differentiation inhibition and excessive apoptosis induced by LPS in chicken embryonic myogenic cells (Figure 9). This study will provide a theoretical basis for future efforts to improve the growth performance of poultry by adding PAMK to feed.

## AUTHOR CONTRIBUTIONS

Yunmao Huang and Xumeng Zhang conceived and designed the experiments. Mengsi Fu, Fada Li, Zhiyuan Liu, Yong Li and Chenyu Zhu performed the experiments and wrote the paper, and Jinhui Wang, Danning Xu, Nan Cao and Wanyan Li helped interpret the results. All authors have read and agreed to the published version of the manuscript.

## CONFLICT OF INTEREST STATEMENT

All authors declare no conflicts of interests.

### ETHICS STATEMENT

None.

### PEER REVIEW

The peer review history for this article is available at https://publons.com/publon/10.1002/vms3.1412.

## Data Availability

Data sharing not applicable to this article as no datasets were generated or analysed during the current study.
